# Spots All Over: Records of Piebaldism in Red‐Tailed Squirrel *Syntheosciurus granatensis* in Northwestern Ecuador

**DOI:** 10.1002/ece3.73969

**Published:** 2026-07-08

**Authors:** Elias Viteri‐Basso, Chiara Correa‐Zanotti, Mateo Roldan, Carlos Morochz, Edward Powers, Bastian Betancourt, Santiago Molina, Rebecca Zug

**Affiliations:** ^1^ Laboratorio de Carnívoros Universidad San Francisco de Quito Quito Ecuador; ^2^ Paris Lodron Universität Salzburg Salzburg Austria; ^3^ Departamento de Investigación y Biología Mashpi Lodge Quito Ecuador

**Keywords:** camera trapping, citizen science, neotropics, pigmentary disorder, sciuridae

## Abstract

Pigmentation anomalies such as albinism, leucism, and melanism in mammals are uncommon but can provide insight into genetic, ecological, and demographic patterns within populations. We documented piebaldism, a form of leucism, in *Syntheosciurus granatensis* using camera‐trap bycatch data (2014–2024), verified iNaturalist observations, and records from collaborators in northwestern Ecuador. From camera‐trap data, 96 of 301 independent events (31.9%) showed piebald individuals. We also compiled 36 iNaturalist observations and five additional records from collaborators. Piebald and normally colored individuals (uniformly colored reddish‐brown coat with bright, rust‐colored tail) occurred sympatrically, with depigmentation patterns ranging from small white patches to extensive white coloration. The records occurred from 70 to 3200 m.a.s.l. in both protected and disturbed areas. These results indicate a high prevalence of piebaldism in this area and demonstrate the value of integrating camera trapping and citizen science data to document color variation.

## Graphical abstract

Color variations in mammals are uncommon but can provide insight into genetic and ecological patterns within populations. We documented a high prevalence of piebaldism in *Syntheosciurus granatensis* in northwestern Ecuador using camera‐trap bycatch (2014–2024), verified iNaturalist observations, and collaborator records, with 31.9% of all independent camera‐trap events showing piebald individuals. Piebald patterns varied from small white spots to extensive depigmentation across a broad elevational range (70–3200 m.a.s.l.), raising questions about the genetic, demographic, and environmental factors driving this unusually high frequency of color variation.

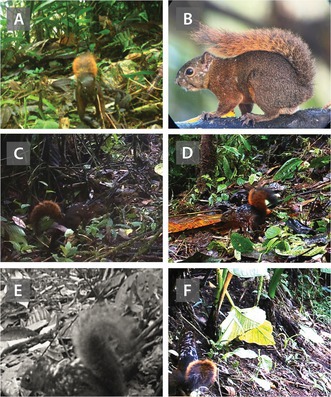

## 1 Introduction

The red‐tailed squirrel *Syntheosciurus granatensis* is a small sciurid distributed from Costa Rica to northern Peru and Venezuela, including several northern South American islands (Heaney and Thorington 1978; Nitikman 1985; Thorington Jr et al. 2012; Tirira 2017; Castro and Borgo 2021). It is one of the most studied species of neotropical squirrels (Mendes et al. 2019), and a large part of its ecology is known. They are diurnal, principally arboreal, solitary animals known to be seed predators but are also important seed dispersers for a variety of plants, including palms and various fruiting trees (Bonaccorso et al. 1980; Glanz 1984; Becker et al. 1985; Carvajal and Adler 2008; Tirira 2017). They occupy small, sometimes overlapping home ranges across all forest levels and can even forage on the ground. They can be found in primary, secondary, and heavily disturbed forests as well as tree plantations (Heaney and Thorington 1978; Carvajal and Adler 2008; Thorington Jr et al. 2012; Castro and Borgo 2021). In Ecuador, this species typically exhibits reddish‐brown dorsal pelage with a rufous to rust‐colored underbelly and tail (Tirira 2017). Once treated as *Sciurus granatensis* , as with most squirrel species, it has undergone various systematic updates. A common synonym used for the species is the use of the genus *Notosciurus*, now considered a lower synonym of the recognized genus *Syntheosciurus* (de Abreu‐Jr et al. 2020).

While color variations such as lighter or darker coats, or slightly different patterns, can be common within some species of squirrels (Thorington Jr et al. 2012), evidence of genetic conditions that affect pigmentation, such as leucism and albinism, is scarce. Despite extensive ecological knowledge of the species, rare phenotypic traits remain poorly documented, likely because they are difficult to detect through traditional field methods. However, camera trapping has shown to be an effective, non‐invasive tool for observing and monitoring wildlife, especially elusive species and in dense, remote ecosystems (Trolliet et al. 2014; Caravaggi et al. 2017; Delisle et al. 2021). Camera traps facilitate the collection of data on behavior, diet, breeding, habitat use, activity patterns, and other aspects of species ecology (O'Connell et al. 2011; Meek et al. 2015; Murphy et al. 2018) and can also record rare phenotypic conditions such as color variations and unusual coloration patterns (Tobler et al. 2008; Aximoff, Hübel, et al. 2021; Guastalla et al. 2021; Viteri‐Basso et al. 2024). Different concentrations of melanin in vertebrates are linked to color variations, including melanism, albinism, leucism, and piebaldism (Pawelek and Körner 1982; Abreu et al. 2013). Piebaldism, in particular, is the partial lack of melanin and is expressed as white or yellowish spots or patches on areas of the body or fur (Pawelek and Körner 1982). Color variations are mainly caused by genetic mutations that affect tyrosinase, a melanin regulator (Hubbard et al. 2010; Lindgren et al. 2015). These conditions occur across a wide range of vertebrate groups but are rare within them (Abreu et al. 2013; Caro and Mallarino 2020). Most color variations have been constantly attributed to small population size, low genetic pool, or ecological factors such as forest cover related to light filtration (Hubbard et al. 2010; Lindgren et al. 2015; Mooring et al. 2020).

Here we report cases of piebaldism in individuals of *Syntheosciurus granatensis* in northwestern Ecuador using evidence from camera‐trap bycatch data and citizen‐science observations.

## 2 Materials and Methods

We documented cases of piebaldism in red‐tailed squirrels in our focus area by compiling photo and video records from three different sources: camera trap projects conducted by the co‐authors within and north of the Mashpi Tayra Wildlife Refuge (MTWR), public records from the citizen science tool iNaturalist, and data points shared from other camera trap projects in the same region of northwestern Ecuador. Squirrels with piebaldism were identified as individuals that exhibited clearly white spots, spotting, or white parts of the body (Pawelek and Körner 1982).

### 2.1 By‐Catch Data From Mammal‐Focused Camera Trap Studies

The majority of the data used for this analysis came from our mammal‐focused camera trap studies in tropical humid forests in northwestern Ecuador, both within the MTWR (0°09′55″ N 78°52′45″ W) from 2014 to 2024 and to the north, on privately owned lands between the MTWR and Cotacachi‐Cayapas National Park (0°33′08.3″N 78°36′41.4″W) from 2019 to 2020. This area of northwestern Ecuador is one of the most biodiverse regions globally (Stattersfield 1998), with an extremely wet climate, hilly terrain (Jahn 2011; Opitz‐Stapleton 2016; Newell et al. 2026), and facing increasing anthropogenic pressures from agriculture, grazing, mining, and urbanization (González‐Jaramillo et al. 2016; Buck et al. 2020). Records of piebald squirrels were obtained as camera‐trap bycatch from four independent projects conducted in this area between 2011 and 2024. These projects employed a range of sampling approaches, including both informal and opportunistic camera trapping as well as systematic survey designs. Two projects (2014–2021 and 2017–2021) involved non‐systematic, opportunistic wildlife monitoring along trails with variable camera‐trapping effort. Whereas the two later projects (2019 and 2023–2024) used systematic camera‐trap surveys, placing camera traps in 2 km^2^ and 1 km^2^ grid cells, respectively, to assess mammal communities, habitat connectivity, and large carnivore ecology. Across all four studies, 74 cameras trap stations were monitored for a total 8256 trap nights. Most camera trap stations consisted of one camera set at no higher than knee height and camera traps were checked every 2 months, on average. Camera trap records were processed using the Timelapse software (Saul Greenberg, University of Calgary) (Greenberg et al. 2019), where all squirrels were identified and piebald records extracted. We considered “observations” as independent events, or hits, defined as consecutive records of the same species at the same camera trap station within a 60‐min interval (Oliveira‐Santos et al. 2013; Gracanin et al. 2022). Red‐tailed squirrels were identified based on their medium to big size and chestnut‐brown reddish to red coloration that distinguishes them from all other squirrels in the region (Tirira 2017). We then calculated the proportion of independent events of piebald squirrels out of all independent events of red‐tailed squirrels across all camera traps from these projects.

### 2.2 iNaturalist Records

Next, we searched for observations showing similar phenotypic features from squirrels in Ecuador on the iNaturalist platform (http://www.inaturalist.org, California Academy of Sciences, and National Geographic Society), filtering by the species (in this case recognized on the platform as *Sciurus granatensis* ) and by country (Ecuador). The search was undertaken on the 2nd of November 2025. These observations were added to the project we created, “Piebald Squirrels” (https://www.inaturalist.org/projects/piebald‐squirrels). Only individuals of this species in Ecuador that exhibited piebaldism and had geolocation accuracy of less than 3 km were considered and included in the project before the records were downloaded for analysis.

### 2.3 Data Points From Other Camera Trap Projects

In addition, we inquired through informal networks if other records of this anomaly had been recorded in the greater study area, in similar forest habitats in northwestern Ecuador. Records confirmed with photographic evidence were included in the study.

## 3 Results

**FIGURE 1:**
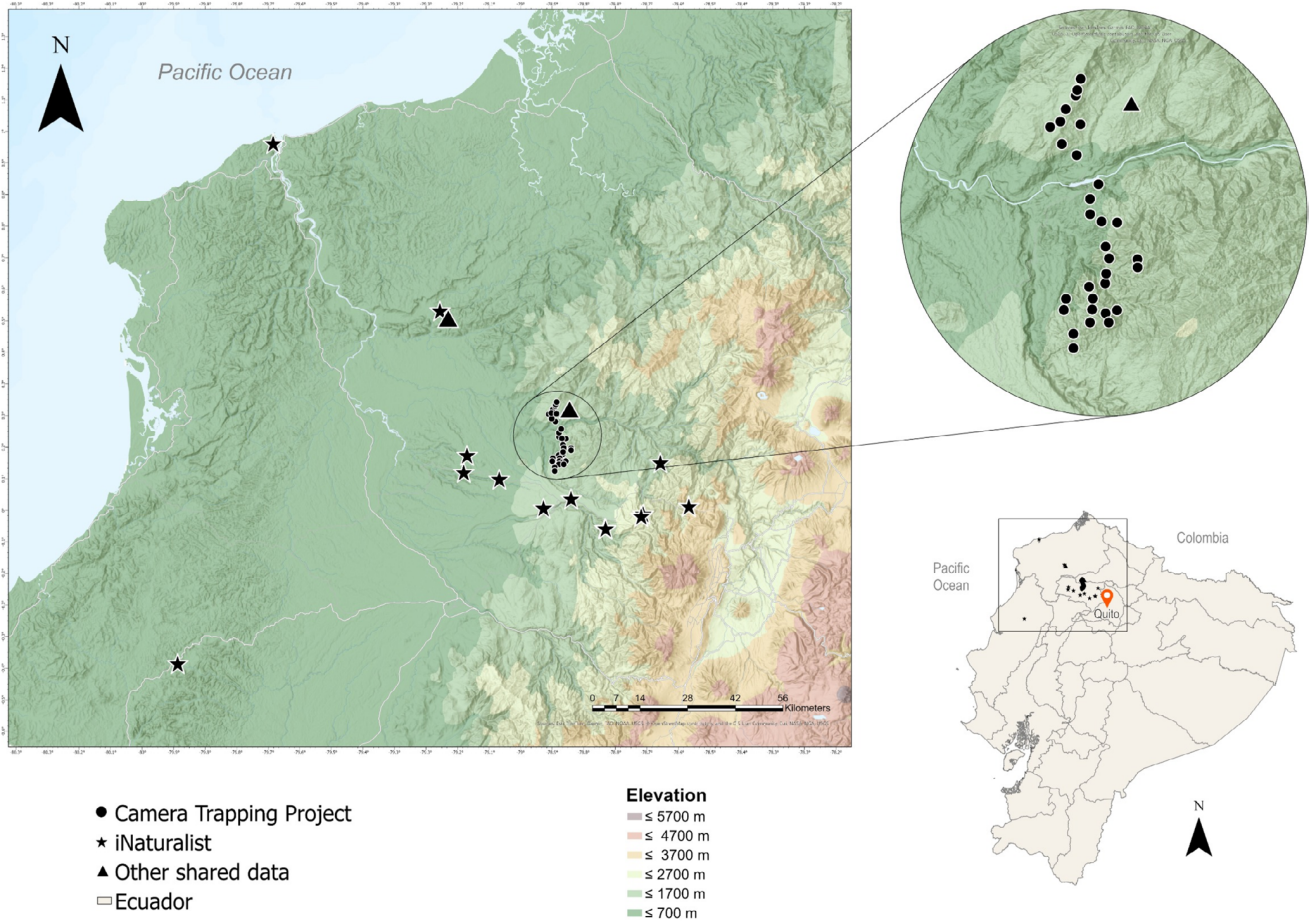
Records of piebaldism in *S. granatensis* in northwestern Ecuador. Records from different camera trap projects conducted within and north of the MTWR (black dots and top right zoom‐in); verified iNaturalist records (black stars), and data points shared from other camera trap projects in the region (black triangles). A total of 137 independent records were obtained in all the study from these sources.

In total, 137 records of piebald squirrels were obtained for this study (Figure 1). Of these, 96 independent events (hits) of squirrels with piebaldism were recorded from our camera trap projects within and north of the MTWR. These events represent 31.89% of the 301 total red‐tailed squirrel detections across all camera trap studies in this category.

A total of 36 observations of piebald squirrels in Ecuador were obtained from iNaturalist. Five additional records from four different sites (Tandayapa Cloud Forest Station, Bellavista Cloud Forest Reserve, Reserva Canandé, and Reserva Río Manduriacu) were collected from other camera trap projects.

**FIGURE 2:**
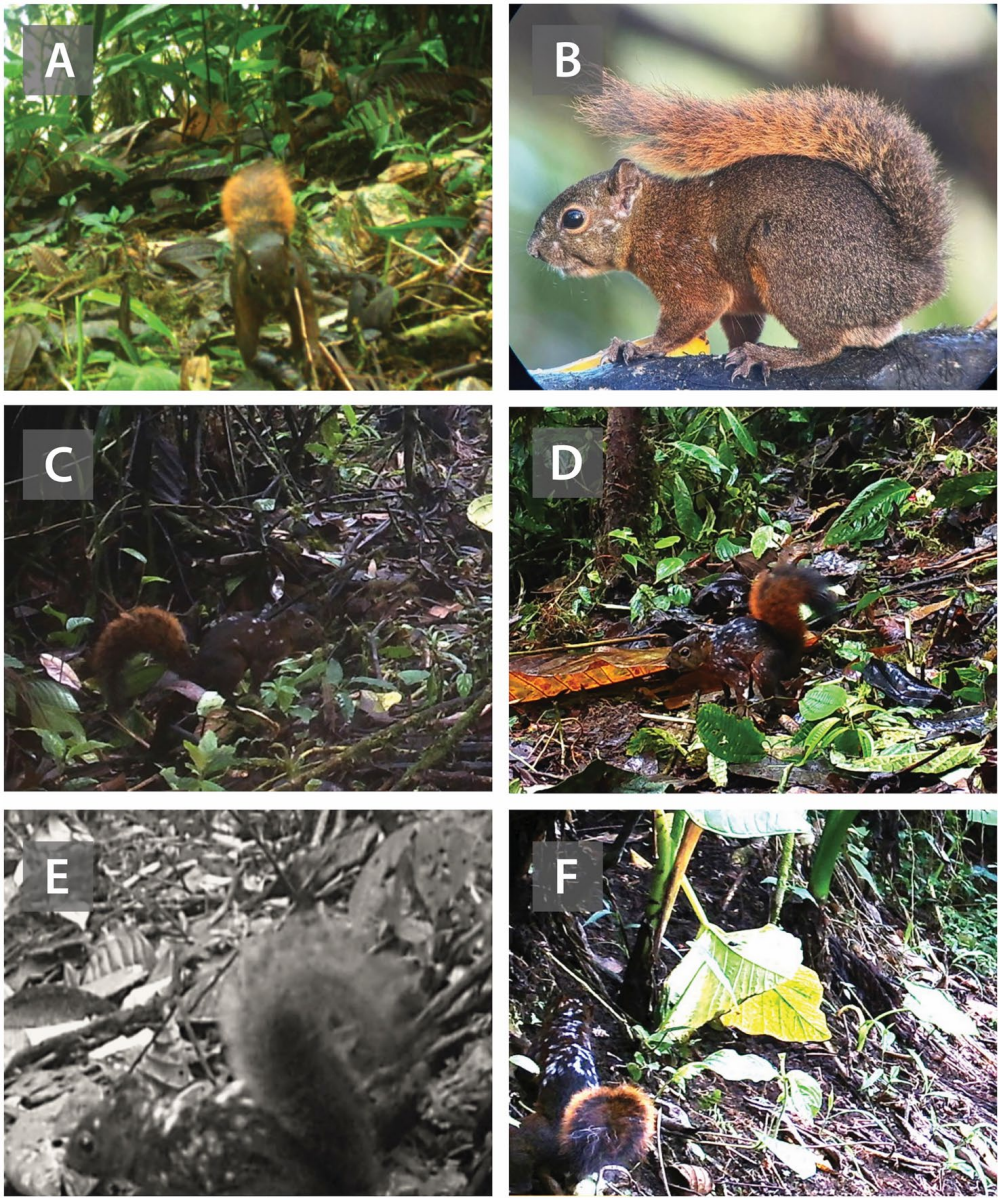
Examples of piebaldism observed in squirrels in northwestern Ecuador. (A) Squirrel with little to no spotting‐ 3 spots (camera trap record). (B) Squirrel with some spotting localized on head and very few on flanks (picture taken by R. Zug). (C) Squirrel with moderate spotting on flanks and back (camera trap record). (D) Squirrel with high levels of spotting on back, flanks, legs, and head (camera trap record). (E) Squirrel with high levels of spotting on back, flanks and head (camera trap record). (F) Squirrel with heavy spotting on back, flanks, and head (camera trap record).

Piebaldism in the squirrels in this study was typically expressed as small white dots in various forms (Figure 2), including variations in the location (spots in the head, flanks or back, or tail); in the quantity or frequency of spots (one singular spot, some spotting, to higher and condensed spotting over parts of body); and in the shape of the spotting (dots, marks, tip of tail). Most piebald features were localized on the head, back, and upper flanks. Two records showed a squirrel with the tip of the tail completely white, one from our camera trap projects and another from an iNaturalist observation.

## 4 Discussion

Color variation in squirrels, including albinism, leucism, and piebaldism, has been reported for many species across mostly the arboreal families such as Callosciurinae, Ratufinae, and Sciurinae (Lajo‐Salazar et al. 2021). For the Americas, all three conditions have been described for seven squirrel species, mainly in Canada and the United States (Mearns 1898; Dunn 1921; Wood 1922, 1965; Jones 1923; Keith 1965; Stencel and Ghent 1987; Jung and Slough 2012; McCardle 2012; Ferron and Laplante 2013; Lajo‐Salazar et al. 2021), but only two species with these conditions are reported from South America: a leucistic Brazilian squirrel *Guerlinguetus brasiliensis* from Rio de Janeiro, Brazil (Tavares et al. 2019), a piebald red‐tailed squirrel *Syntheosciurus granatensis* in El Oro, Ecuador (Ramírez‐Jaramillo 2019), and an albino *S. granatensis* from a collection in Cauca, Colombia (Ramírez‐Chaves et al. 2008). The latter observations may indicate that the prevalence of color alterations in *S. granatensis* is not limited to our observations in northwestern Ecuador, showing alterations across its range (S Colombia and S Ecuador).

Although relatively uncommon, some studies report high proportions of individuals exhibiting color variations. Documented cases of abnormal coloration include five albino eastern gray squirrels *Sciurus carolinensis* in central Texas (McCardle 2012), at least 20 populations of albino eastern gray squirrels nationwide in the United States (Guiles 1997), and a colony of leucistic eastern fox squirrels *Sciurus niger* in Austin, Texas (Weber and Weber 2012). In contrast, most other studies report only isolated individuals or pairs displaying color abnormalities.

The high proportion of piebald squirrels in a population may indicate a limited genetic pool (Hubbard et al. 2010; Mooring et al. 2020; Caro and Mallarino 2020), environmental pressures such as diet, habitat, and ecological patterns (Caro 2005; Lamoreux et al. 2010), or could be the result of an adaptive, positive trait that has been successfully passed on through generations (Abreu et al. 2013; Schneider et al. 2015; Viteri‐Basso et al. 2024). In this case, the appearance of piebaldism represented as white spotting on the body does not seem to favor any positive condition, and there does not appear to be any evidence of favorable traits linked to the spots (Caro 2005; Aximoff, Hübel, et al. 2021; Funkhouser et al. 2019; Whitehead et al. 2025).

Instead, there may be negative ecological implications of piebaldism in this population. The condition may alter cryptic patterns or camouflage coloration, impacting their ability to hide from predators and therefore making them more prone to predation (Simpson 1994; Caro 2005; Goode and Pasachnik 2016; Aximoff, Hübel, et al. 2021; Bleich 2024). However, we cannot exclude the possibility that sunlight filtering through the canopy creates a dappled light pattern that may enhance camouflage in piebald individuals. It may also influence social interactions and reproduction, especially in individuals with high proportions of piebaldism (Goode and Pasachnik 2016; Aximoff, Neto, et al. 2021; Whitehead et al. 2025), and it may impact other health‐ and fitness‐related processes, including implications from oxidative stress and melanocyte migration associated with ontogenic development (Vafaee et al. 2010; Funkhouser et al. 2019; Whitehead et al. 2025). Finally, as mentioned previously, the high prevalence of piebaldism recorded may be the result of low genetic diversity within the larger population, potentially driven by restricted habitat area and limited connectivity, that may have resulted in inbreeding and the persistence of this genetic trait (Hubbard et al. 2010; Mooring et al. 2020; Caro and Mallarino 2020).

It is important to note that due to the camera trap configuration, all observations were restricted to the forest floor and likely underestimated the true abundance of squirrels, and therefore the proportion of piebald individuals in the area. It is also worth mentioning that hits of piebald squirrels do not translate to the number of individuals, based on their site fidelity and small range size.

However we do interpret the high prevalence of piebaldism in these red‐tailed squirrels as a population‐level pattern rather than an anomaly restricted to a few individuals. This inference is supported by the diversity of unique spotting patterns observed and the large area covered by camera traps and iNaturalist records in which affected individuals were recorded, particularly given the species' site fidelity and limited home range size. Notably, piebald squirrels were sympatric with normally colored individuals; in several cases, both phenotypes were recorded at the same camera trap station, sometimes on the same day. This raises interesting questions regarding the interactions between the two. In addition, piebaldism was observed on both sides of the major river as Guayllabamba, and across an altitudinal gradient (70–2150 m.a.s.l.), suggesting that the trait is not geographically isolated. Additional research regarding landscape connectivity and gene flow is needed to draw further conclusions.

It is important to further understand the causes and effects of the prevalence of piebaldism in the study area, including whether similar patterns occur in other species or are linked to local environmental or demographic factors with potential conservation relevance. We highlight the need for additional studies on color variation in tropical vertebrate communities, as well as the value of citizen science platforms such as iNaturalist for documenting and compiling rare phenotypic observations. We understand that our records are not the only occurrences of this condition for the country and invite other researchers to collaborate by sharing and consolidating records of piebald squirrels and other species exhibiting abnormal coloration patterns, particularly in regions characterized by restricted species ranges and high habitat fragmentation.

## Author Contributions

**Elias Viteri‐Basso:** conceptualization (lead), data curation (lead), formal analysis (lead), methodology (lead), writing – original draft (lead), writing – review and editing (equal). **Chiara Correa‐Zanotti:** investigation (supporting), visualization (supporting), writing – review and editing (supporting). **Mateo Roldan:** investigation (supporting), writing – review and editing (supporting). **Carlos Morochz:** investigation (supporting), writing – review and editing (supporting). **Edward Powers:** investigation (supporting), writing – review and editing (supporting). **Bastian Betancourt:** investigation (supporting), writing – review and editing (supporting). **Santiago Molina:** investigation (supporting), writing – review and editing (supporting). **Rebecca Zug:** conceptualization (supporting), investigation (lead), project administration (equal), resources (lead), supervision (lead), visualization (supporting), writing – review and editing (equal).

## Conflicts of Interest

The authors declare no conflicts of interest.

## Data Availability

The data supporting the findings of this study are publicly available in Zenodo: https://doi.org/10.5281/zenodo.20820261.
